# Impact of Bead-Beating Intensity on the Genus- and Species-Level Characterization of the Gut Microbiome Using Amplicon and Complete 16S rRNA Gene Sequencing 

**DOI:** 10.3389/fcimb.2021.678522

**Published:** 2021-10-01

**Authors:** Bo Zhang, Matthew Brock, Carlos Arana, Chaitanya Dende, Nicolai Stanislas van Oers, Lora V. Hooper, Prithvi Raj

**Affiliations:** ^1^ Department of Immunology, University of Texas Southwestern Medical Center, Dallas, TX, United States; ^2^ Microbiome Research Laboratory (MRL), Department of Immunology, University of Texas Southwestern Medical Center, Dallas, TX, United States

**Keywords:** microbiome, 16S, sequencing, DNA extraction, bead-beating, short-read, long-read

## Abstract

Bead-beating within a DNA extraction protocol is critical for complete microbial cell lysis and accurate assessment of the abundance and composition of the microbiome. While the impact of bead-beating on the recovery of OTUs at the phylum and class level have been studied, its influence on species-level microbiome recovery is not clear. Recent advances in sequencing technology has allowed species-level resolution of the microbiome using full length 16S rRNA gene sequencing instead of smaller amplicons that only capture a few hypervariable regions of the gene. We sequenced the v3-v4 hypervariable region as well as the full length 16S rRNA gene in mouse and human stool samples and discovered major clusters of gut bacteria that exhibit different levels of sensitivity to bead-beating treatment. Full length 16S rRNA gene sequencing unraveled vast species diversity in the mouse and human gut microbiome and enabled characterization of several unclassified OTUs in amplicon data. Many species of major gut commensals such as *Bacteroides, Lactobacillus, Blautia, Clostridium, Escherichia, Roseburia, Helicobacter*, and *Ruminococcus* were identified. Interestingly, v3-v4 amplicon data classified about 50% of *Ruminococcus* reads as *Ruminococcus gnavus* species which showed maximum abundance in a 9 min beaten sample. However, the remaining 50% of reads could not be assigned to any species. Full length 16S rRNA gene sequencing data showed that the majority of the unclassified reads were *Ruminococcus albus* species which unlike *R. gnavus* showed maximum recovery in the unbeaten sample instead. Furthermore, we found that the *Blautia hominis* and *Streptococcus parasanguinis* species were differently sensitive to bead-beating treatment than the rest of the species in these genera. Thus, the present study demonstrates species level variations in sensitivity to bead-beating treatment that could only be resolved with full length 16S rRNA sequencing. This study identifies species of common gut commensals and potential pathogens that require minimum (0-1 min) or extensive (4-9 min) bead-beating for their maximal recovery.

## Introduction

Trillions of symbiotic microbial cells are present in and on the human body that constitute human microbiota (Huttenhower et al., 2021). The microbiome refers to the collection of genes in these microorganisms. Specific clusters of these microbes in various body parts constitute the organ-specific microbiome, for example microbial communities in the gut constitute the gut microbiome. The microbiome includes a variety of organisms, i.e., bacteria, yeasts, fungi, protozoa, and viruses (Matijašić et al., 2020). However, bacteria comprise the vast majority of these microorganisms which play a critical role in the breakdown and absorption of nutrients, sugars, and proteins that humans cannot otherwise digest and metabolize on their own to synthesize essential amino acids and vitamins. Microbiome profiling assays typically sequence polymorphisms in the 16S rRNA gene of bacteria, the 18S rRNA gene of eukaryotes, and ITS regions in the case of fungi to infer taxonomic classification of the microbiome (Janda and Abbott, 2007; Nash et al., 2017). Literature suggests that DNA extraction methods significantly impact the microbiome study results (Costea et al., 2017; Sinha et al., 2017). Many studies have optimized protocols to extract microbial DNA from different types of samples to use as a template for 16S rRNA gene sequencing (Gill et al., 2006; Nelson et al., 2010; Werner et al., 2012; Falony et al., 2016; The Integrative HMP (iHMP) Research Network Consortium, 2019). A number of prior studies provide evidence that methods of sample collection, storage, and DNA extraction are critical for accurate profiling of microbiota in environmental (Baker et al., 2003; Tremblay et al., 2015; Bag et al., 2016) or human samples (Wu et al., 2010; Momozawa et al., 2011; Willner et al., 2012; Brooks et al., 2015; Costea et al., 2017; Sinha et al., 2017). Literature suggests that complete lysis of the bacterial cell wall is critical for optimum yield of high integrity DNA for both short and long-read sequencing workflows (Jenkins et al., 2019). Lysis protocols include procedures that lead to physical and or enzymatic disruption of the microbial cell wall (Bag et al., 2016; Gill et al., 2016; Valentini et al., 2016). It has been observed that extended lysis time and mechanical disruption can enhance nucleic acid yield. However, extended mechanical lysis time can also reduce molecular complexity by excessive shearing of microbial DNA into smaller fragments (von Wintzingerode et al., 1997; Dilhari et al., 2017). Particularly, Gram-positive bacteria pose the greatest challenge for complete lysis due to their thick cell walls and complex composition (Kim et al., 2015).

Metagenomic analysis of the human microbiome shows that each individual can harbor hundreds of different bacterial species and varying lysis methods can impact their recovery (Qin et al., 2010; Gill et al., 2016). It is therefore very important to use a DNA extraction method that can optimally extract DNA from the entire bacterial community with minimal bias for downstream sequencing analysis. Current DNA extraction methods use various sample homogenization or lysis protocols, which can result in variability shown in microbiome profiles. Mechanical bead-beating or enzymatic cell lysis steps have been shown to be crucial for maximum DNA recovery from all kinds of organisms (de Boer et al., 2010). Bead-beating has become a common method of bacterial cell lysis in microbial metagenomics studies (Fiedorova et al., 2019). Here we assess the impact of bead-beating treatment on gut microbiome recovery using the v3-v4 amplicon and the full length 16S rRNA sequencing method. We characterize genus- and species-level diversity in mouse and human stool and assess variation in OTU recovery pertaining to differential sensitivity to bead-beating treatment in the DNA extraction protocol.

## Materials and Methods

### Sample Collection

We investigated five mouse (C57/Bl6) stool samples (designated as M1-M5), five human stool samples (designated as H1-H5), and one ZymoBIOMICS Gut Microbiome Standard (Cat#D6331) from Zymo Research. The Zymo control sample is comprised of 21 different bacterial and fungal strains that mimic the human gut microbiome. Prior knowledge on the composition and proportions of various bacteria in this sample allowed for validation of our sequencing and data analysis pipeline. Stool samples were collected under sterile conditions and stored in DNA/RNA Shield, a nucleic acid stabilizing solution from Zymo Research (R1100). DNA/RNA Shield provides an accurate molecular signature of the sample at the time of collection by preserving nucleic acids at ambient temperature and inactivating organisms including infectious agents. Human stools were collected from healthy volunteers under UT Southwestern Institutional Review Board (IRB) Number STU-022011-211. All research protocols and experiment methods used in this study were approved by the IRB. All participants gave their written informed consent to participate in the research.

### Bead-Beating Condition and DNA Extraction Method

**Figure 1:**
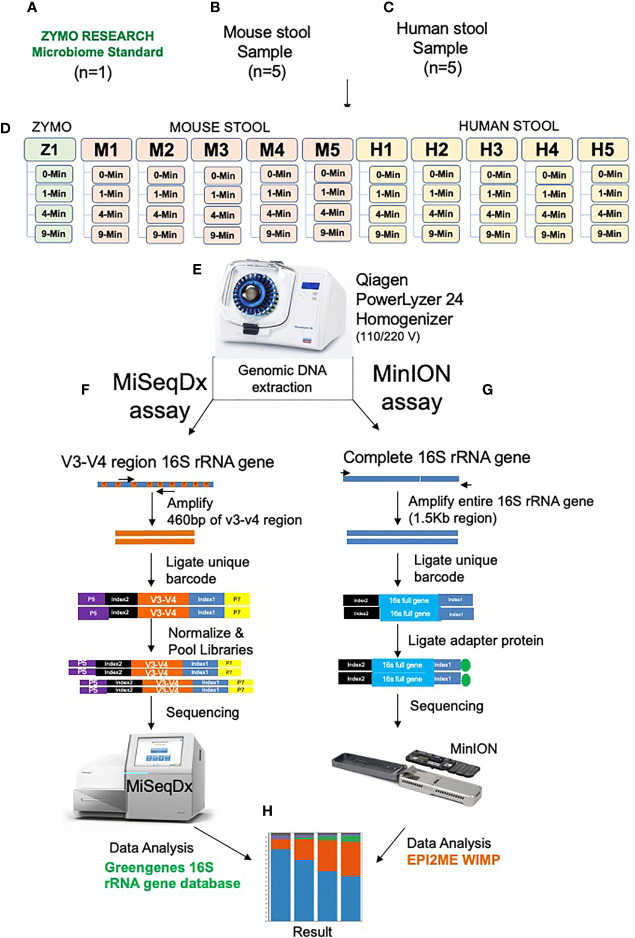
Illustration of the experimental design and workflow. One Zymo human gut microbiome mock control D6331 **(A)**, Five mouse stool samples **(B)**, Five human stool samples **(C)**, Experiment design **(D)**, PowerLyzer 24 Homogenizer used for homogenization **(E)**. Gut microbiome sequencing using V3-V4 16S rRNA amplicon method on MiSeq, Illumina **(F)**, Full length 16S rRNA gene sequencing on MinION, Oxford Nanopore **(G)** and OTU clustering analysis **(H)**.

We used the ZymoBIOMICS™ DNA Miniprep Kit (D4300) for DNA extraction on all the study samples. **Figure 1** illustrates the design and experimental workflow of the study. To make sure that each aliquot received unbiased representation of the sample, the specimen was first hand-mixed using the spoon provided in the DNA/RNA Shield Fecal Collection Tubes. Then once all the large clumps were dissolved in the specimen and the sample appeared to be more uniform in solution, 1 ml of aliquots were prepared for various bead-beating conditions. Similarly, 75 ul of ZymoBIOMICS Gut Microbiome Standard (D6331) and 925 ml of DNA/RNA Shield was aliquoted into each of four separate tubes to test with four bead-beating conditions. Each sample was aliquoted into a ZR BashingBead lysis tube (0.1 and 0.5 mm beads). Next, each sample tube was tightly closed and loaded onto the PowerLyzer 24 Homogenizer (110/220 V) for bead-beating. We selected four different bead-beating time points as illustrated in **Figure 1**: 0 min (no bead-beating at all), 1 min (one cycle of shaking for 1 min), 4 min (two cycles of 2 min shaking, with a 2 min pause after each cycle), and 9 min (6 cycles of 1 min 30 s, with a 2 min pause after each cycle). Each of these samples were bead-beaten at a speed of 2200 RPM and were maintained at a temperature of 20°C throughout the bead-beating process. Following beat-beating and lysis, DNA was purified using the ZymoBIOMICS protocol, and 100 ul was eluted for downstream experiments. The DNA concentration was measured using the Picogreen method (Invitrogen Quant-iT™ Picogreen dsDNA Assay Kit Reference No. P11496 on Perkin Elmer 2030 Multilabel Reader Victor X3), and the DNA integrity number (DIN) was determined on 4150 Tapestation from Agilent using Agilent’s gDNA Screen Tape (Reference No. 5067-5365) and Agilent’s gDNA Reagents (Reference No. 5067-5366).

**Figure 2:**
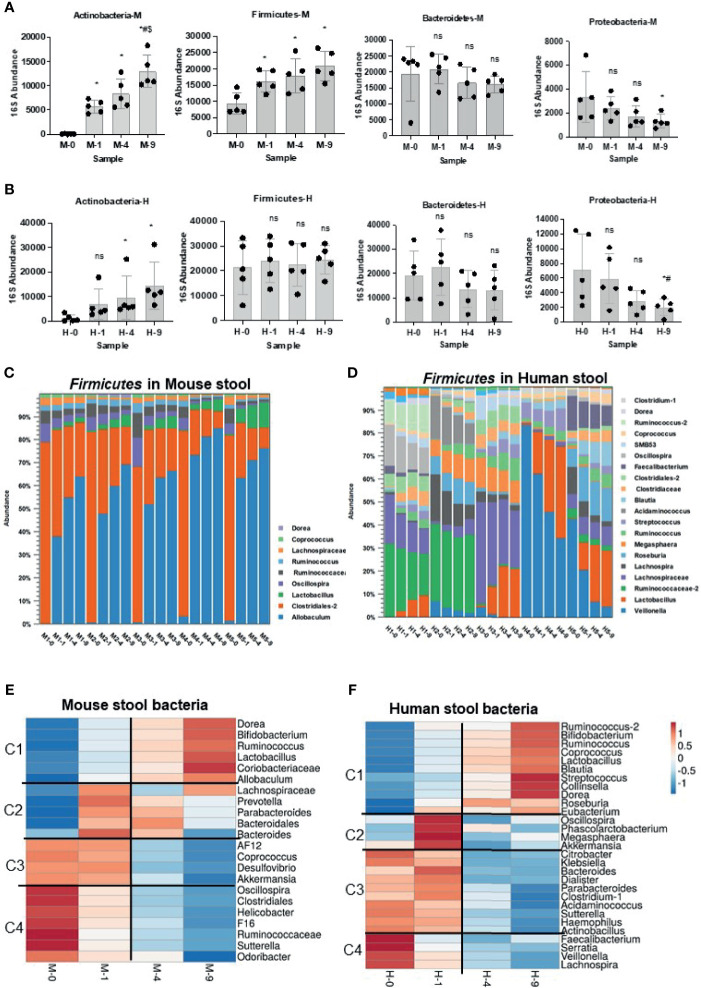
Impact of bead-beating on *Phylum-* and *Genus*-level recovery of gut microbiota. Panel **(A)** Relative abundance of *Actinobacteria, Firmicutes, Bacteroidetes, and Proteobacteria* at four bead-beating conditions in mouse stool. Panel **(B)** shows the relative abundance of *Actinobacteria, Firmicutes, Bacteroidetes, and Proteobacteria* at four bead-beating conditions in human stool. Each black dot represents an individual stool sample. Bar plots show relative 16S abundance on the y-axis and samples on the x-axis. Panels **(C)** shows detected abundances of various *Firmicutes* in mouse stool. Panel **(D)** shows abundances of *Firmicutes* in human stool. Panel **(E)** Heatmap shows four clusters of mouse stool bacteria that differ in their abundances at various bead-beating conditions. Panel **(F)** Heatmap shows four clusters of human stool bacteria that differ in their abundances at various bead-beating conditions. These clusters were generated using *ClustVis*, a web tool for visualizing clustering of multivariate data (BETA) (Metsalu and Vilo, 2015). Blue and red color in the heatmap indicate lowest or highest abundance, respectively. Statistical p-values are denoted with *, ^#^ and ^$^ represent comparison of unbeaten sample data with 1-, 4-, and 9-min beaten samples, respectively. ns, not significant.

### 16S rRNA v3-v4 Amplicon Sequencing on MiSeqDx

We used 10-20 ng of stool-purified DNA to amplify hypervariable region V3-V4 of the bacterial 16S rRNA gene using the Zymo Research Quick-16S NGS Library Prep Kit (Catalog No. D6400). A single amplicon of about 460 bp was amplified using the Quick-16S Primer Set V3-V4 included in the library prep kit. PCR was stopped using the Reaction Clean-Up Solution per the kit’s recommendations. Next, Zymo Research Index Primer Set A was ligated to the amplicon for multiplexing. After the addition of barcodes, the final library size was 596 bp. Then the libraries were then brought to a volume of 50 ul with the addition of DNase/RNase-free water and purified using 56 ul of Beckman Coulter’s AMPure XP beads (Catalog No. A63881). The final libraries were eluted using 27.5 ul of Tris-HCl buffer (10mM, pH 8.5). Quality and quantity of each sequencing library were assessed using Agilent’s 4150 Tapestation using gDNA Screen Tape and gDNA Reagents both from Agilent and picogreen measurements, respectively. The libraries were then pooled in equal concentrations according to picogreen measurements. Each pool was quantified using the KAPA Biosystems Library Quant Kit (illumina) ROX Low qPCR Mix (Reference No. 07960336001) on an Applied Biosystems 7500 Fast Real-Time PCR system. According to the qPCR measurements, 6 pM of pooled libraries was loaded onto a MiSeqDX flow cell and sequenced using MiSeq Reagent Kit v3 600 Cycles PE (Paired end 300 bp). Raw FASTQ files were demultiplexed based on unique barcodes and assessed for quality.

### Full Length 16S rRNA Gene Sequencing on MinION

To determine the impact of bead-beating intensity on species-level diversity in the gut microbiome, the full length 16S rRNA gene was sequenced on subset samples (one Zymo, two Mouse, and three human samples) using long-read sequencing technology from Oxford Nanopore. About 20 ng of DNA was used to amplify and barcode the entire (1.5 Kb) segment of the 16S rRNA gene using Nanopore’s 16S Barcoding Kit (SQK-16S024). The PCR product was then purified using Beckman Coulter’s AMPure XP beads (Catalog No. A63881). The purified amplicon libraries were quantified using the Qubit dsDNA HS Assay kit (Catalog No. Q32854) on an Invitrogen Qubit 4 fluorometer. Then sequencing adapters were ligated to the pooled barcoded reads according to the manufacturer’s instructions. Sequencing was performed using R9.4.1 flow cell for 24 h on the MinION (Oxford Nanopore Technologies). Nanopore sequence data was analyzed with EPI2ME Agent v2020.2.10. The 16S sequences were assigned taxonomy using the What’s in my pot? (WIMP) workflow as illustrated in **Figure 1**.

### Amplicon and Full Length Sequence Data Analysis

Samples with more than 50 K QC pass short sequencing reads from MiSeqDx were used for 16S OTU analysis. Taxonomic classification and operational taxonomic units (OTUs) abundance analysis was performed using the CLC Bio microbial genomics module (https://www.qiagenbioinformatics.com/plugins/clc-microbial-genomics-module/). Individual sample reads were annotated with the Greengenes v13 database using a 97% similarity index. Alpha and beta diversity analysis was done to understand within- and between-treatment group diversity, respectively. Nanopore 16S data were analyzed using the EPI2ME pipeline and WIMP workflow from Oxford Nanopore Technology (ONT). Raw FASTQ files from Illumina and Nanopore sequencing have been deposited in the Sequence Read Archive (SRA) with accession no. PRJNA685188 (v3-v4 amplicon data).

### Data Analysis

**Figure 3:**
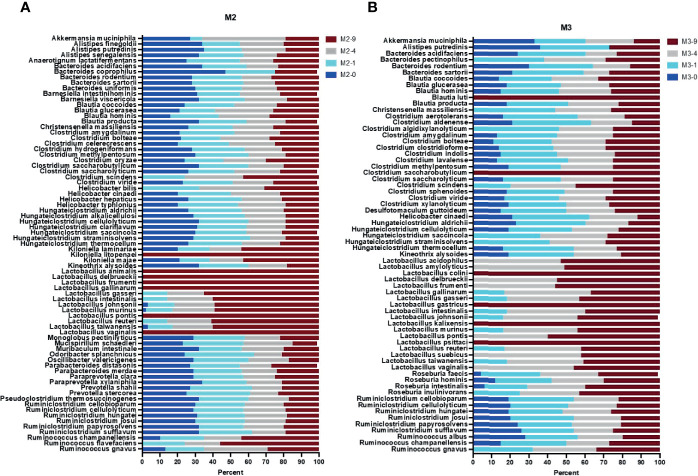
Full length 16S rRNA gene sequence-based taxonomic classification of bacteria in mouse stool samples. Plots in panels **(A, B)** show relative abundance of detected species at four bead-beating conditions in M2 and M3 stool, respectively. The x-axis shows the percentage of reads supporting the taxon and the y-axis shows the detected species. In the sample ID, M represent Mouse and -0, -1, -4, and -9 represent bead-beating time in the experiment.

**Figure 4:**
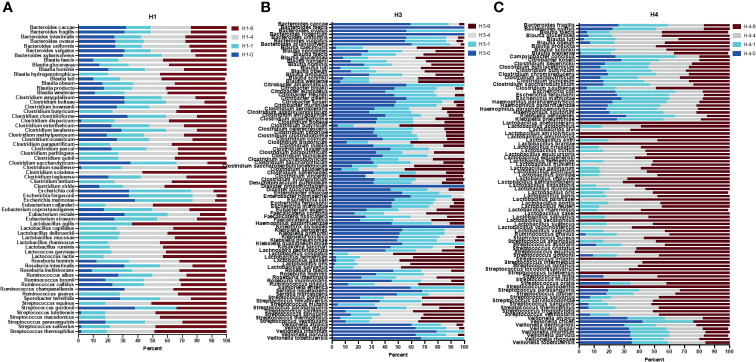
Full length 16S rRNA gene sequence-based taxonomic classification of bacteria in human stool samples. Plots in panels **(A–C)** show relative abundance of detected species at four bead-beating conditions in H1, H3, and H4 stool, respectively. The x-axis shows the percentage of reads supporting the taxon and the y-axis shows detected species. In the sample ID, H represent Human and -0, -1, -4, and -9 represent bead-beating time in the experiment.

**Figure 5:**
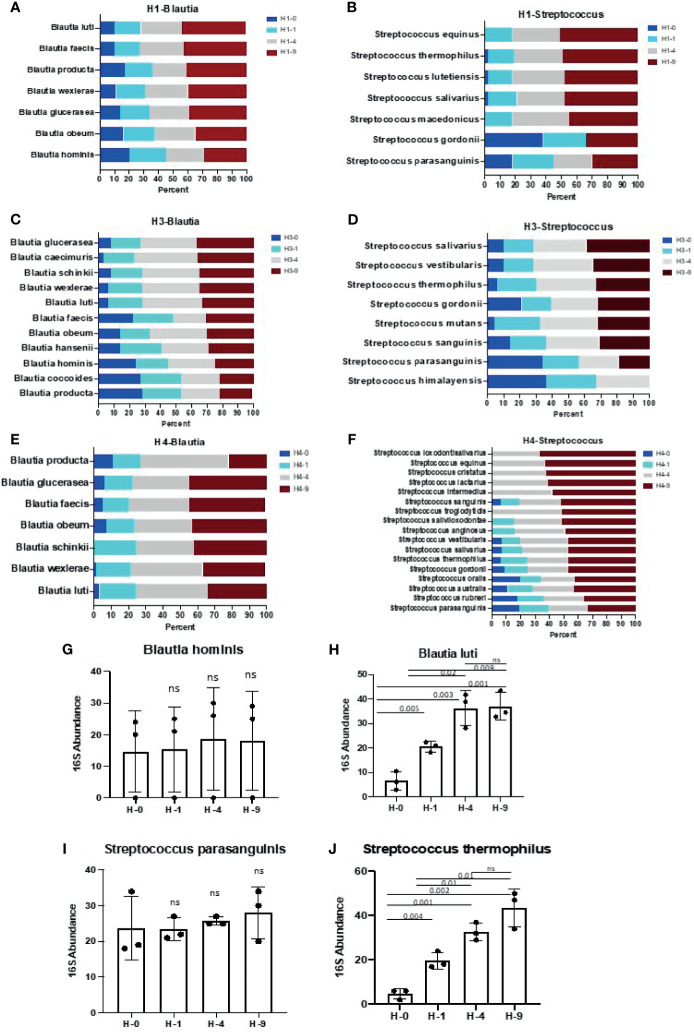
Species of genus *Blautia and Streptococcus* show differential sensitivity to bead- beating treatment. Panels **(A–F)** illustrate the abundance of various species of *Blautia and Streptococcus* at four (0, 1, 4, and 9 min) bead-beating conditions in three human stool samples (H1, H3, H4). Relative abundance of various species of *Blautia and Streptococcus* at each condition is plotted in bar graphs. Relative abundance was calculated based on number of full length 16S rRNA sequencing reads supporting a given taxon in each condition. Panel **(G)** shows abundance of *Blautia hominis* at four different bead-beating conditions in human stool. Panel **(H)** shows abundance of *Blautia luti* at different bead-beating conditions in human stool. Panel **(I)** shows abundance of *Streptococcus parasanguinis* at four different bead-beating conditions in human stool. Panel **(J)** shows abundance of *Streptococcus thermophilus* at different bead-beating conditions in human stool. The y-axis shows the 16S abundance of detected bacteria and the x-axis shows different beating conditions. Numeric data were compared using t-test statistics and a p-value <0.05 was considered significant. “ns” denotes “Not significant”. Sample IDs denotes H-0: 0 min, H-1: 1 min, H-4:4 min, and H-9:9 min of bead-beating time.

To compare the microbiome diversity between samples and treatments, we applied PERMANOVA analysis (PERmutational Multivariate ANalysis Of VAriance, also known as non-parametric MANOVA (Tang et al., 2016) available in CLC Bio Microbial Genomics Module 20.0). This measures effect size and significance on beta diversity for variables. The significance is obtained by a permutation test. In addition, abundances across various bead-beating conditions were compared using a linear model differential abundance test. This tool models each feature (e.g., OUT or an organism) as a separate generalized linear model (GLM). Microbiome compositions were compared across time points using DEseq (DESeq2_1.26.0), in R version 3.6.1. Data in **Figures 3**–**5** represent relative abundance of species determined based on the number of reads detected for that species. Total observed reads for a species were normalized to 1 and then relative abundance at each treatment point was calculated. Statistical analysis was performed using GraphPad Prism software version 8 (GraphPad). T-test was performed and a two-tailed p value of <0.05 was considered significant. MinION sequencing data were analyzed using EPI2ME Agent v2020.2.10; 16S sequencing reads were assigned taxonomy using the WIMP workflow.

## Results

### Impact of Bead-Beating Intensity on DNA Integrity and Quantity

As shown in **Supplementary Figure 1**, total DNA yield was significantly (t-test p<0.05) high in the samples beaten for 4 and 9 min (**Supplementary Figure 1A**). DNA integrity number (DIN) was high in samples beaten for 1 or 4 min (**Supplementary Figure 1B**). The number of pass filter sequencing reads were similar across the treatments (**Supplementary Figure 1C**). We also compared the total number of high-confidence OTUs annotated in all the samples in the amplicon and full length sequencing data. Median values of these data are compared using a t-test and presented in scatter plots in **Supplementary Figure 1D**. Overall, the data suggest that bead-beating has no significant impact on the number of sequence reads or OTUs (**Supplementary Figure 1D**).

### Impact of Bead-Beating Intensity on Microbial Diversity

Quality pass sequencing reads were used to cluster OTUs in the study samples. **Supplementary Figure 2** shows v3-v4 amplicon data-based phylum level OTUs in mouse and human stool samples. Alpha and beta diversity indices were determined for various bead-beating intensities (**Supplementary Figures 3A, B**). As shown in **Supplementary Figure 3B**, mouse samples were tightly clustered based on bead-beating condition. The Bray-Curtis method of beta-diversity assessment was used to evaluate dissimilarity index between communities. Shannon’s entropy and Simpson’s indices were reduced upon extensive bead-beating (**Supplementary Figures 3C, D**). However, overall phylogenetic diversity was higher in 4-min bead-beaten mouse stool (**Supplementary Figure 3E**). Abundance of OTUs in different bead-beating treatments was compared using generalized linear model differential abundance test on groups defined by bead-beating treatments (**Supplementary Table 1**). In addition, PERMANOVA analysis was also used to measure significance on beta diversity (**Supplementary Table 2**). Similar analysis was performed in human stool data and OTUs with statistically different abundances (**Supplementary Table 3**). **Supplementary Figures 3F, G** show alpha- and beta diversity analysis in human stool samples. As shown, beta-diversity indices in human stool samples were very different from that of mice, as higher between sample diversity was observed (**Supplementary Figure 3G** and **Supplementary Table 4**). We observed high *Simpson’s index* and *Shannon entropy* as well as phylogenetic diversity in 4- and 9-min bead-beaten samples in human stool (**Supplementary Figures 3H–J**), respectively. Overall, analysis showed that studied parameters were more consistent for the 4 min group in both mouse and human samples.

### Validation of Sequencing and Analysis Pipeline on Mock Control

We tested and standardized our 16S sequencing and data analysis pipeline on a Zymo mock control sample. As shown in **Supplementary Figure 4** and **Supplementary Table 5**, our assay was able to recover all Gram-negative and Gram-positive strains in a mock sample in very similar proportions as pooled by Zymo Research (Pearson r=0.75-0.87, p<0.001), suggesting that our assay was capturing the read out quite accurately. As expected, bead-beating had a relatively moderate impact on the recovery of Gram-negative bacteria such as *Escherichia, Prevotella*, and *Akkermansia* (**Supplementary Figure 4A**). On the other hand, maximum abundance of Gram-positive bacteria such as *Roseburia, Bifidobactarium, and Lactobacillus* was only captured either in 4- or 9-min beaten samples (**Supplementary Figure 4B**). Consistent with literature that suggests that the complex cell wall in Gram-positive bacteria requires more intense lysis, we observed a strong correlation (Pearson’s r =0.91) between bead-beating intensity and recovery of Gram-positive strains in mock control (**Supplementary Figure 4C**). We used the same sequencing protocol and analysis pipeline to analyze the gut microbiome in mouse and human stool next.

### Bead-Beating Treatment Stratifies Gut Microbes Into Four Major Clusters

Analysis of 16S rRNA v3-v4 amplicon sequencing data in mouse stool showed that the recovery of phylum *Actinobacteria and Firmicutes* was significantly (p<0.05) affected by bead-beating intensity (**Figure 2A**). As illustrated, maximum recovery of *Actinobacteria* and *Firmicutes* was observed in samples beaten for 4 and 9 min. On the other hand, the highest abundance of *Proteobactaria* and *Bacteroidetes* was observed in unbeaten or 1-min beaten samples (**Figure 2A**). Consistent with mouse stool data, *Actinobacteria, Bacteroidetes, and Proteobacteria* in human stool also showed similar sensitivity to bead-beating treatment, however human samples were more heterogeneous (**Figure 2B**). Interestingly, *Firmicutes* detected in mouse and human stool exhibited different sensitivity to bead-beating treatment (**Figures 2C, D**). As shown in **Figure 2C**, *Firmicutes* in mice stool were mostly comprised of *Allobaculum* and *Clostridiales* and showed a consistent pattern of recovery across all the samples (**Supplementary Table 6A**). On the other hand, *Firmicutes* in human stool were comprised of more diverse bacteria that showed large sample-to-sample heterogeneity in composition and abundances (**Figure 2D**). As shown, *Allobaculum* in mouse stool required extensive bead-beating for maximum recovery, whereas *Firmicutes* in human stool, i.e., *Veillonella, Ruminococcus*, and *Acidaminococcus* showed maximal recovery in unbeaten or 1-min beaten samples (**Supplementary Table 6B**). These differences in the composition of *Firmicutes* in mouse and human stool were the reasons for discordant results. Analysis of top bacteria in mouse stool data showed that recovery of *Lactobacillus*, *Allobaculum, Bifidobacterium, Coriobacteriaceae, F16*, and *Clostridiales* was significantly (p<0.05) affected by bead-beating treatment (**Supplementary Table 7**). Similarly, comparison of OTUs in human stool samples revealed the differences in abundances between the four bead-beating conditions (**Supplementary Table 8**). Analysis showed that recovery of *Dorea, Blautia, Ruminococcus, Lactobacillus*, and *Bifidobacterium* was significantly (p<0.05) affected by bead-beating intensity (**Supplementary Table 8**). These data suggest that applying the same bead-beating treatment to mouse and human stool samples may obscure the actual diversity of the gut microbiome.

Next, we clustered the genus-level OTUs in mouse and human stool based on their sensitivity to bead-beating intensity. We used ClustVis (Metsalu and Vilo, 2015) to stratify bacteria into clusters based on their abundance at various bead-beating conditions (**Figures 2E, F**). As shown in **Figure 2E**, this analysis stratified the top 22 OTUs into four major clusters in mouse stool. Cluster 1 was comprised of bacteria *Dorea, Bifidobacterium, Lactobacillus, and Allobaculum* that showed maximum abundance in the 9-min beaten sample as compared to the unbeaten sample. The second cluster included bacteria such as *Prevotella* and *Bacteroides* that showed maximum recovery at 1 or 4 min of bead-beating. Cluster 3 included bacteria that required minimal shaking, 1 min of or no bead-beating at all. The fourth cluster was comprised of organisms such as *Helicobacter* and *Sutterella* that showed maximum recovery in the unbeaten sample (**Figure 2E**).

Similar clusters of bacteria were also observed in human stool as well (**Figure 2F**). Cluster 1 included many of the common human gut commensals such as *Dorea, Blautia, Bifidobacterium*, and *Lactobacillus*. These organisms were very underrepresented in unbeaten samples. Cluster 2 constitutes the moderate group that certainly required some (1 min) bead-beating treatment as suggested by its reduced recovery in both 0 as well as in 9-min beaten samples. Interestingly, cluster 3 included some known human pathogens such as *Klebsiella, Hemophilus*, and *Citrobacter.* These organisms showed maximum recovery in unbeaten or 1-min beaten samples (**Figure 2F**). Cluster 4 showed maximum recovery of OTUs in unbeaten samples. This cluster included *Faecalibacterium, Serratia, Veillonella*, and *Lachnospira* (**Figure 2F**).

### Impact of Bead-Beating Intensity on the *Species*-Level Recovery of the Gut Microbiome

The 16S v3-v4 amplicon sequencing data were very limited in species-level taxonomic classification of detected OTUs (**Supplementary Table 9**). About 35-40% of OTUs were classified up to a species level of taxonomy. The remaining 60-65% were only classified up to the phylum, class, order, family, or genus level (**Supplementary Table 10**). So, next we performed full length 16S rRNA gene sequencing on two mice (M2, M3) and three human stool samples (H1, H2, H4). Long-read sequencing data was classified into various taxonomic ranks using the EPI2ME WIMP pipeline. NCBI taxonomy trees were generated based on the number of detected reads. The 16s v3-v4 amplicon and full length 16S rRNA sequencing data showed good correlation at the phylum level (Pearson r>0.70, p<0.01). Full length data characterized vast species-level diversity in mouse and human samples (**Figures 3**, **4**). The 16S v3-v4 amplicon data annotated 14 and 10 OTUs to the species level of taxonomy in M2 and M3 mouse stool, respectively. On the other hand, full length sequences-based analysis revealed 98 and 96 bacterial species in M2 and M3 mouse stool, respectively. Similarly, amplicon data in H1, H3, and H4 samples annotated 10, 11, and 8 OTUs to species level respectively, whereas, full length sequence analysis assigned species rank to 155, 143, and 120 detected organisms in H1, H3, and H4 samples, respectively. These numbers were calculated based on a minimum of 15 long reads to support a taxon. Analysis of full length data showed that about 78% (75 out of 97) of the total observed species in M2 could be detected in the unbeaten sample, whereas only 59% (56 out of 95) of the total observed species were detected in the unbeaten M3 stool, suggesting potential differences in the proportion of bacteria that are more sensitive to bead-beating treatment. Next, to calculate the relative percentage of observed species at each time point, we normalized the total reads for the given species to 100 percent. Data presented in **Figures 3**–**5** show normalized relative percentages of observed species. This allowed comparison of recovery in terms of observed abundance of the species at each bead-beating treatment. Furthermore, analysis showed that about 27% (26 out of 95) of bacteria in M3 stool showed maximum abundance in 4- or 9-min beaten samples as compared to 10% (10 out of 97) in M2 (**Figure 3B** and **Supplementary Figure 5**). As shown in **Supplementary Figure 5**, long-read sequencing data profiled species diversity in several major gut commensal genera, i.e., *Bacteroides, Clostridium, Lactobacillus, Ruminococcus*. We observed that some species in *Roseburia, Blautia, and Ruminococcus* showed variation in their recovery with respect to bead-beating treatment. Of these, *R*. *gnavus* and *R. albus* species of the *Ruminococcus* genus were particularly interesting as the maximum abundance of *R*. *gnavus* was detected in 4- and 9-min beaten samples, whereas *R. albus* species showed maximum abundance in 0- or 1-min beaten mouse samples (**Supplementary Figure 5H** and **Supplementary Table 11**).

Next, full length 16S rRNA sequences-based microbiome analysis in three human stool samples (H1, H3, and H4) revealed vast species diversity (about an 8-10-fold increase compared to amplicon data) and sample-to-sample variation in the microbiome composition as suggested by variable number and types of detected bacteria (**Figure 4** and **Supplementary Table 12**). The number of bacteria detected in different bead-beating treatments varied from sample to sample (**Supplementary Table 12**). It was observed that species of the genus *Blautia*, *Streptococcus, and Ruminococcus* exhibited variation in recovery with respect to bead-beating treatment (**Supplementary Figures 6A–E**). Full length sequencing data analysis showed significant diversity and heterogeneity in organisms detected in human stool (**Figure 4B**). For example, in sample H3, some clinically relevant microbes such as *Citrobacter freundii and Klebsiella pneumoniae* were also detected (**Supplementary Figures 6F–J**). We observed the highest abundance of these bacteria in the unbeaten sample (**Supplementary Figures 6F–J**). Similarly, species of *Lactobacillus* and *Streptococcus* in the H4 sample consistently showed highest abundance in the 9-min beaten sample (**Supplementary Figures 6K–N**). Although three samples are not enough for statistical comparisons, these data do explore microbial diversity and sample-to-sample heterogeneity in microbiota composition in human fecal material. Many OTUs in H1 and H4 showed maximum abundances in 4-9-min beaten samples. On the other hand, a large number of OTUs in H3 stool showed maximum abundance in the unbeaten sample (**Figure 4** and **Supplementary Table 12**). Next, we sorted bacterial species based on Gram-positive and Gram-negative classification and assessed their recovery across four bead-beating conditions in H3 stool. As expected, Gram-negative species showed maximum abundance in the unbeaten sample (**Supplementary Figure 7**). We compared amplicon OTU composition in mouse and human stool across bead-beating time points using DEseq analysis (**Supplementary Tables 13**, **Supplementary Table 14**). We also compared abundances of various species detected in full length 16S data analysis across four bead-beating treatments and identified those that showed significant variation in recovery between four conditions (**Supplementary Table 15**). As shown in **Figures 5A–F**, we observed variation in recovery of *Blautia* and *Streptococcus* species. For example, the abundance of *Blautia hominis* species showed no significant difference in recovery between the four bead-beating conditions, whereas *Blautia luti* and other species of this genus showed maximum abundance in the 9-min beaten sample (**Figures 5G, H** and **Supplementary Table 15**). Similarly, abundance of *Streptococcus parasanguinis* species was not significantly different at the four different bead-beating conditions as was the case with *Streptococcus thermophilus* and other common species of this genus that exhibited significantly (p<0.01) higher abundance in the 9-min beaten sample (**Figures 5I, J**, **Supplementary Table 15**). These data support our hypothesis that different species of a genus may exhibit variation in sensitivity to bead-beating treatment.

### Impact of Bead-Beating on the Recovery of *Ruminococcus gnavus vs. Ruminococcus albus*

**Figure 6:**
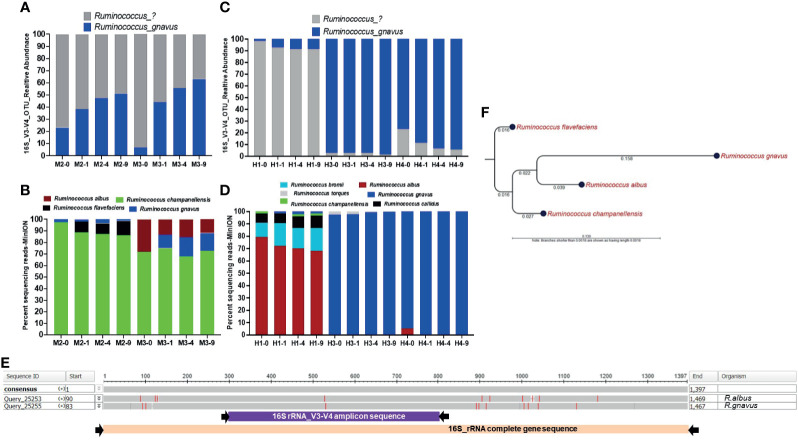
Bead-beating intensity impacts recovery of *Ruminococcus species.* Panel **(A)** Bar plots show abundance of *Ruminococcus* in v3-v4 amplicon data in mouse stool. Panel **(B)** shows full length 16S rRNA sequencing data in mouse stool. Panel **(C)** Bar plots show v3-v4 amplicon data-based abundance of *Ruminococcus* in human stool. Panel **(D)** shows the proportion of various *Ruminococcus species* detected by full length 16S rRNA sequencing data in human stool. The x-axis shows the sample number and four different bead-beating conditions 0 min, 1 min, 4 min, and 9 min. The y-axis for panels **(B, D)** shows the percentage of full length 16S rRNA sequencing reads that support the detected taxon in the 16S analysis EPI2ME-WIMP pipeline. Panel **(E)** Nucleotide sequence alignment on the 16S rRNA gene sequence of *Ruminococcus albus* and *Ruminococcus gnavus* using NCBI’s BLASTn program. The presented image was generated from Multiple Sequence Alignment Viewer 1.20.0. The grey horizontal bar represents conserved 16S rRNA gene sequences and red lines indicate mismatches or differences between two sequences. Purple and pink bars underneath show v3-v4 amplicon and full length 16S rRNA primer-captured gene regions, respectively. Panel **(F)** 16S rRNA gene sequence-based maximum likelihood phylogenetic tree. Complete 16S rRNA gene sequence of *Ruminococcus albus*, *Ruminococcus gnavus, Ruminococcus champanellensis*, and *Ruminococcus flavefaciens* were aligned and the maximum likelihood phylogenetic tree was constructed using CLC Genomic workbench 12.0.2. Numbers on the tree indicate branch length calculated based on the number of substitutions.

In mouse stool data, we observed that about 50% of *Ruminococcus* reads were annotated as *R. gnavus* that showed maximum abundance in 9-min beaten samples in all the mice samples. The remaining reads that could not be assigned any species showed relatively higher abundance in unbeaten or 1-min beaten samples, as shown in M2 and M3 (**Figure 6A**). Full length 16S rRNA sequencing in M2 and M3 samples revealed other species such as *Ruminococcus champanellensis*, *Ruminococcus flavefaciens*, and *Ruminococcus albus* that were rather more abundant than *Ruminococcus gnavus* (**Figure 6B**). Interestingly, we observed that abundance of *Ruminococcus albus* species in M3 long-read data was highest in the unbeaten sample. Similarly, amplicon data on human stool also showed a lot of *Ruminococcus* reads that were not classified into any species, consistent with mouse stool data. Although maximum abundance of *Ruminococcus* was captured in 9-min beaten samples, abundance of unclassified species was also observed at 1 and 4 min as well. The most interesting sample was H1, in which about 80% of *Rumnicoccus* reads could not be classified into any species, whereas the other two samples (H3 & H4) showed a high abundance of *R. gnavus* reads (**Figure 6C**). Full length 16S rRNA gene sequencing in these three samples further revealed the species-level diversity, especially in the H1 sample. As shown in **Figure 6D**, the H1 sample showed the presence of various *Ruminococcus* species including *Ruminococcus albus*, *Ruminococcus bromii*, *Ruminococcus callidus*, *Ruminococcus champanellensis* as well as *Ruminococcus gnavus.* More interestingly, consistent with M3 mouse data, the abundance of *Ruminococcus albus* in the H1 sample was also highest in the unbeaten sample (**Figure 6D**). Unlike H1, samples H3 and H4 were mostly enriched with *Ruminococcus gnavus* species, which is consistent with their amplicon data in **Figure 6C**. Next we performed multisequence alignments and phylogenetic analysis of various *Ruminococcus* species to explore the 16S gene regions of divergences between these species (**Figures 6E, F**). As shown, complete 16S gene sequence alignment between *R. albus* and *R. gnavus* species showed that most of the differences lay outside the v3-v4 hypervariable region (**Figure 6E**). Maximum likelihood phylogenetic analysis on complete 16S rRNA gene sequences showed a genetic relationship between four common *Ruminococcus* species (**Figure 6F**).

### Impact of Bead-Beating Intensity on the Recovery of Known Human Pathogens

To assess the impact of bead-beating on the recovery of some clinically relevant microbes, we performed a bead-beating experiment on the ZymoBIOMICS microbial community standard (D6300) that represents a balanced mixture of common infectious microorganisms including *Listeria monocytogenes, Bacillus subtilis, Staphylococcus aureus, Enterococcus faecalis, Lactobacillus fermentum, Salmonella enterica, Escherichia coli, and Pseudomonas aeruginosa.* The v3-v4 amplicon sequencing analysis on this sample showed that *Bacillus, Listeria, and Lactobacillus* bacteria certainly required 4-9 min of bead-beating for maximal recovery (**Supplementary Figures 8A–C**). On the other hand, *Salmonella, Pseudomonas, and Enterococcus* showed maximum recovery in the unbeaten samples (**Supplementary Figures 8D–F**). The presented data are from two independent experiments on the same Zymo control DNA. Similarly, we also observed a variation in the recovery of clinically relevant microbes in human stool samples. As shown in **Supplementary Figures 8G–J**, *Streptococcus, Dorea, Blautia, and Coporocuccus* exhibited a variation in recovery with respect to bead-beating treatment. It was observed that abundance of these bacteria was significantly (p=0.05) higher in the 9-min beaten sample as compared to the unbeaten sample (**Supplementary Figures 8G–J**). On the other hand, *Hemophilus* and *Citrobacter* showed an opposite trend with maximum recovery in the unbeaten sample (**Supplementary Figures 8K–N**). However, these results are exploratory and need validation in larger sample cohorts in the future.

## Discussion

Accurate assessment of microbiome structure and composition is very important to study the role of gut microbiota in health and disease (Duvallet et al., 2017). Multiple factors including methods of sample collection, sample storage, DNA extraction, sequencing library preparation, and bioinformatics analysis have been shown to contribute to final microbiome results (Cardona et al., 2012; Carroll et al., 2012; Gorzelak et al., 2015; Rintala et al., 2017; Penington et al., 2018; Proctor et al., 2019). Published literature documents the standards and guidelines for processing and analyzing fecal samples for reproducible microbiome analysis (Santiago et al., 2014). Our assessment of the impact of bead-beating treatment on v3-v4 amplicon and full length 16S rRNA sequencing-based analysis of the microbiome reveals the spectrum of species that require minimum or extensive beating for maximum recovery. Observed higher DNA yield and species diversity in 4- and 9-min beaten samples are consistent with published literature (Lim et al., 2018; Teng et al., 2018). Observed maximal recovery of *Actinobacteria* and *Firmicutes* in samples subjected to bead-beating for 9 min is consistent with published reports that show enhanced nucleic acid recovery from Gram-positive organisms with longer disruption of the bacterial cell wall (Yuan et al., 2012). Interestingly, data from the present study showed that bacteria in the mouse and human gut have quantitative variation in sensitivity to bead-beating treatment, as supported by the presence of four different clusters of bacteria. These results suggest that an optimum beating time is necessary to profile the community diversity in a given sample. As also reported by other investigators, our data showed that full length 16S rRNA gene sequencing provides high resolution species-level information on gut microbiota (Johnson et al., 2019; Matsuo et al., 2021), which was not achieved with v3-v4 amplicon sequencing. Our analysis suggests that in general various species of a genus show similar sensitivity to bead-beating intensity. However, some species of *Ruminococcus*, *Blautia, Streptococcus*, *Clostridium, and Roseburia*, do seem to exhibit some variability in sensitivity to beating treatment. However, investigations on pure isolates will be needed to validate these observations in future studies. Results in *Ruminococcus* bacteria are particularly interesting as data suggest that *R. albus* species are more sensitive to bead-beating as compared to *R. gnavus.* Given that *Ruminococcus species* are ubiquitous members of the mammalian gastrointestinal tract and play an important role in the digestion of a wide range of plant cell wall polysaccharides, the observed findings are interesting. Furthermore, our study also shows that v3-v4 data were not able to identify *Ruminococcus albus* species at all. It was full length 16S rRNA data that revealed the significant abundance of *R. albus* in the M3 and H1 samples. Precise species-level identification of OTUs is very important as different species may have very different interactions and impact on the host. For example, *Ruminococcus albus* is a major cellulose degrader in the human gut (Christopherson et al., 2014) and plays an important role in metabolism, on the other hand, blooms of *Ruminococcus gnavus* has been implicated in autoimmune and inflammatory conditions (Henke et al., 2019). Multiple sequence alignment of all four species of Ruminococcus show that *R. albus* and *R. gnavus*, though part of the same clade, are genetically distinct (**Figure 6F**). More interestingly, complete 16S gene sequence alignment between these two species showed that most of the differences lay outside the v3-v4 hypervariable region, which may be the reason that v3-v4 amplicon data could not detect *R. albus* species or distinguish between the two species (**Figure 6E**). Despite the small sample size, the present study demonstrates the advantage of full length 16S rRNA gene sequencing for gut microbiome characterization. Present study data also show that full length 16S rRNA gene sequencing can precisely characterize common and rare species in mouse and human gut microbial communities. Published studies suggest that hundreds of species can co-exist in an individual (Almeida et al., 2019; Yang et al., 2020). So, application of an appropriate DNA extraction method, especially bead-beating intensity, is critical for accurate and comprehensive assessment of species diversity in a sample. In summary, our study demonstrates that the duration of bead-beating has a strong impact on the recovery of common gut commensals as well as clinically relevant microbiota. Our data suggest that a minimum of 4 min of bead-beating (using Qiagen PowerLyzer) can result in recovery of about 70% of gut microbiota. This study stratifies bacterial species in mouse and human stool that require minimum (0-1 min) or extensive (4-9 min) bead-beating for their maximal recovery.

## Data Availability Statement

The datasets presented in this study can be found in online repositories. The names of the repository/repositories and accession number(s) can be found in the article/**Supplementary Material**. 16S sequencing data (raw FASTQ files) and associated metadata have been deposited in NCBI SRA database with accession no. PRJNA685188.

## Ethics Statement

Human stools were collected from healthy volunteers under UT Southwestern Institutional Review Board (IRB) Number STU-022011-211. All research protocols and experiment methods used in this study were approved by the IRB. All participants gave their written informed consent to participate in the research.

## Author Contributions

BZ and MB performed the experiments, CA performed quality control sequencing, CD provided the mouse stool for the study, NO and LH contributed to manuscript editing, and PR conceived and designed the experiments and wrote the manuscript. All authors contributed to the article and approved the submitted version.

## Funding

This study was supported by the UT Southwestern Microbiome Research Laboratory.

## Conflict of Interest

The authors declare that the research was conducted in the absence of any commercial or financial relationships that could be construed as a potential conflict of interest.

## Publisher’s Note

All claims expressed in this article are solely those of the authors and do not necessarily represent those of their affiliated organizations, or those of the publisher, the editors and the reviewers. Any product that may be evaluated in this article, or claim that may be made by its manufacturer, is not guaranteed or endorsed by the publisher.
