# Supplementary material for: Impact of Bead-Beating Intensity on the Genus- and Species-Level Characterization of the Gut Microbiome Using Amplicon and Complete 16S rRNA Gene Sequencing
Source: Front Cell Infect Microbiol. 2021 Oct 1;11:678522. doi: 10.3389/fcimb.2021.678522 (PMC8517478; doi:10.3389/fcimb.2021.678522)
Supplement: Supplementary file 1 [file DataSheet_1.pdf]

Supplementary Figure1: Impact of bead beating intensity on Fecal DNA yield, DNA integrity, sequencing reads and number of OTUs

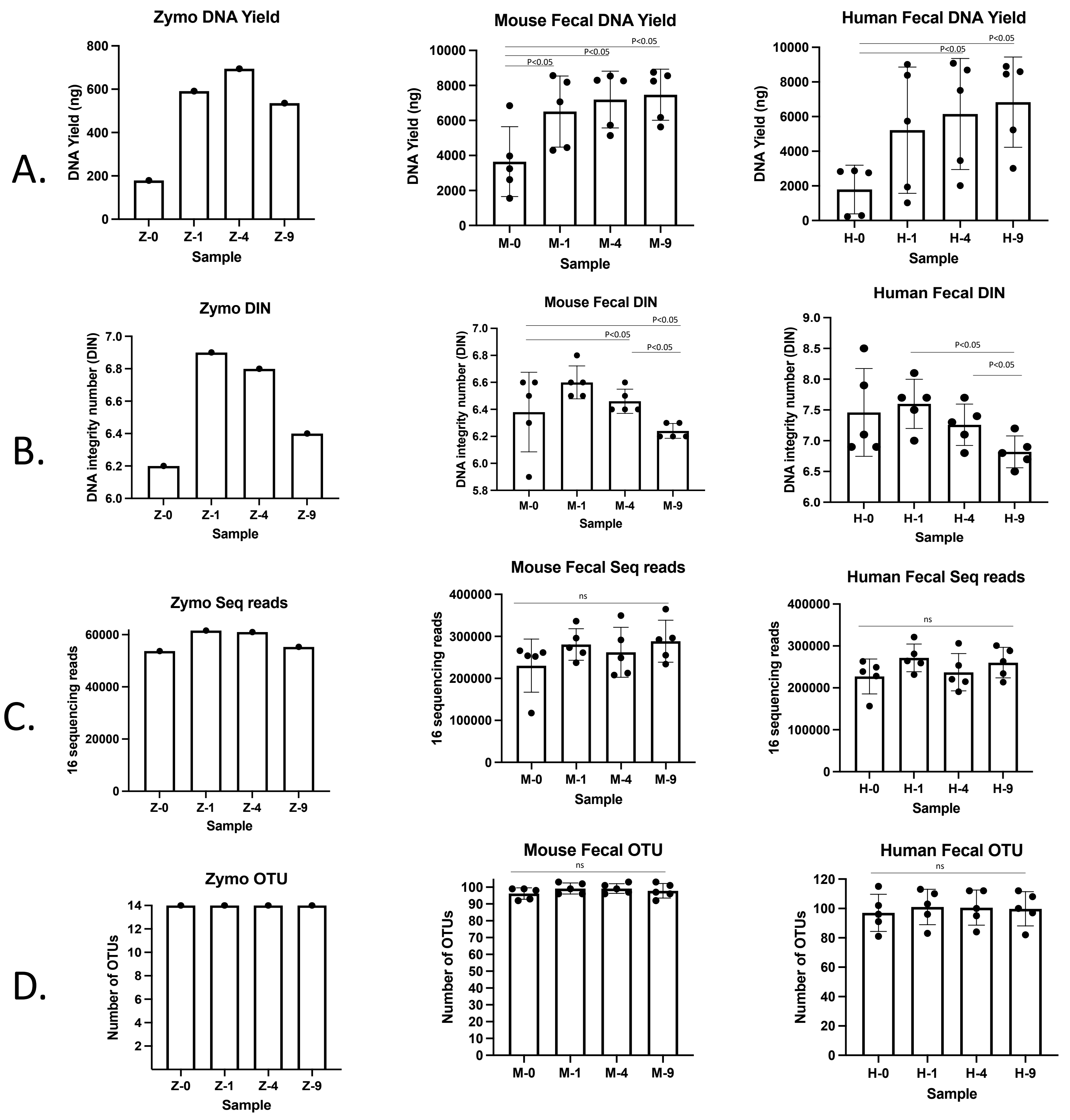

Supplementary Figure 1: Impact of bead beating intensity on total DNA yield, DNA integrity, sequencing reads and number of detected OTUs. "ns" represent not significant p-value. The line in the data plots indicate median value. T-TEST p-values of <0.05 are only shown.

Supplementary Figure2: Phylum level OTU clustering in mouse and human stool- v3-v4 amplicon data

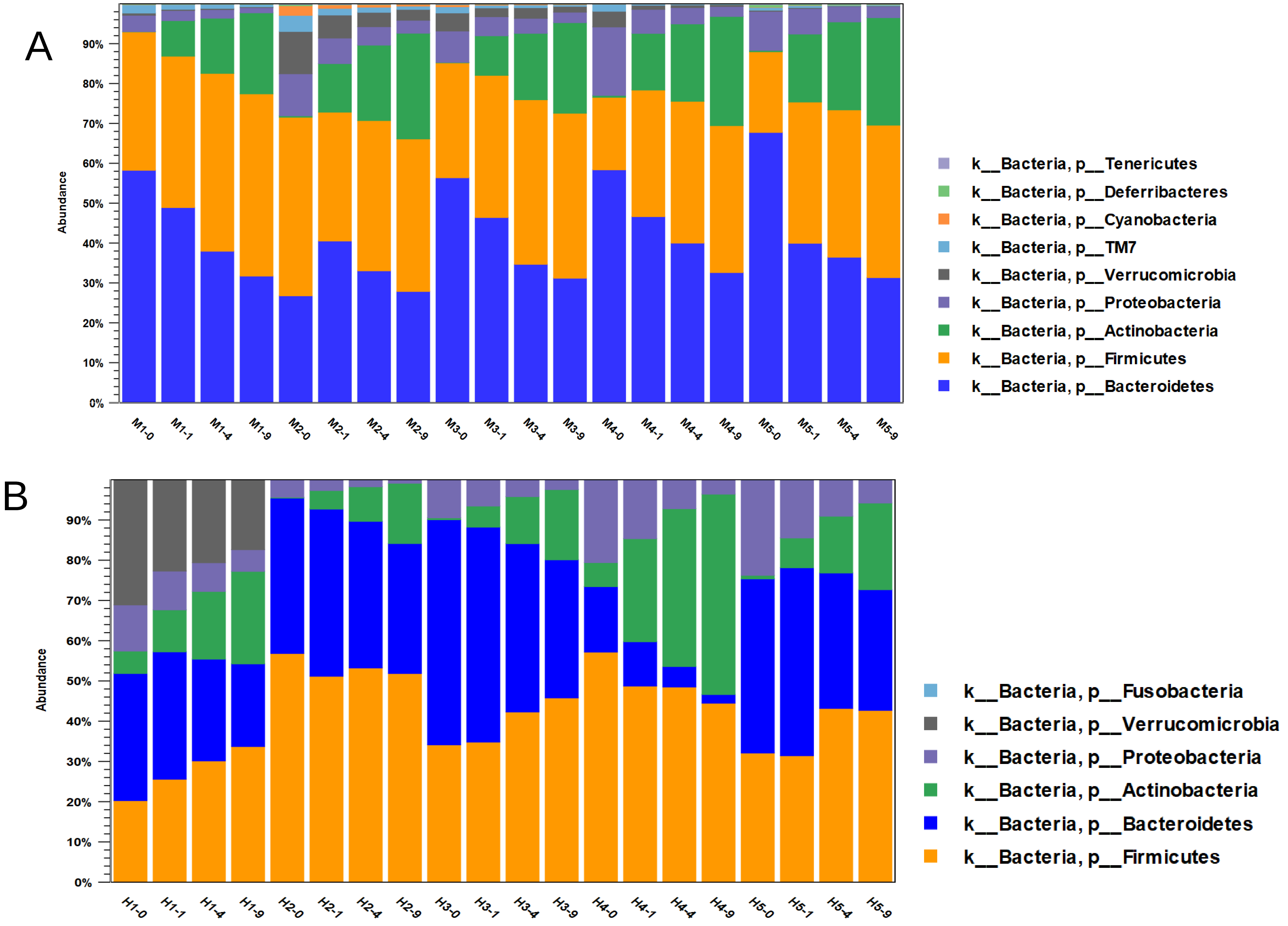

Supplementary Figure 2: Panel A show 16S v3-v4 amplicon sequences inferred phylum level OTUs in five Mouse stool samples**.** Each bar represent individual sample and various color represent relative proportion of detected bacteria. Panel B show 16S v3-v4 amplicon sequences inferred phylum level OTUs in five Human stool samples. Each bar represent individual sample and various color represent relative proportion of detected bacteria. M1, M2, M3, M4, M5 denote five animals. H1, H2, H3, H4, H5 denote five individuals. -0, -1, -4 & -9 indicate bead-beating treatment minutes. X-axis show samples and y-axis show relative abundances.

Supplementary Figure 3: Alpha, Beta-diversity, *Shannon's entropy, Simpson's index* and phylogenetic diversity in study samples

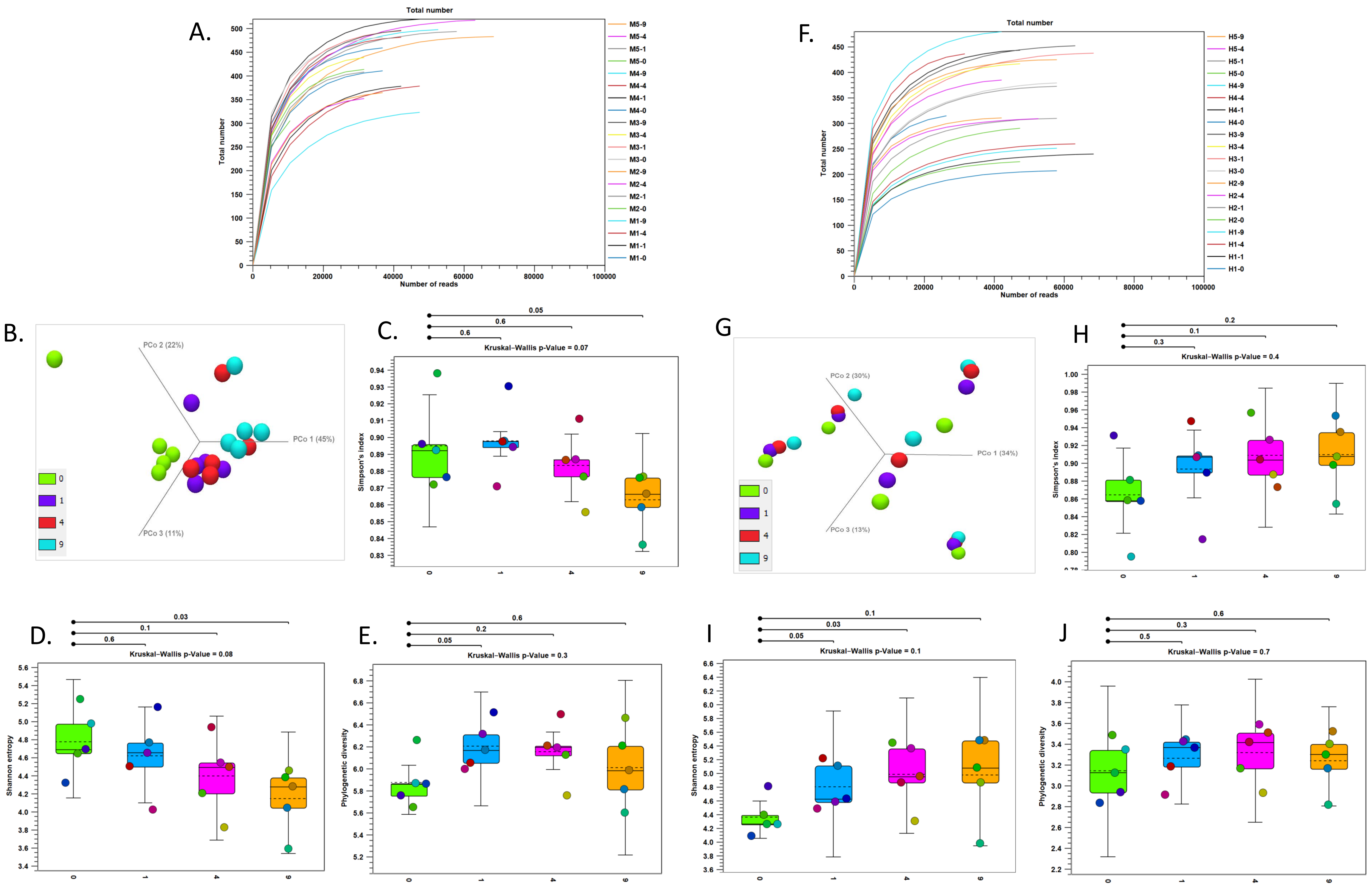

Supplementary Figure 3: Alpha, Beta-diversity, *Shannon's entropy, Simpson's index* and phylogenetic diversity based on v3-v4 rRNA amplicon sequencing data. Presented analyses are from 16s v3-v4 amplicon sequencing data. Panel A-E present mouse stool data. Panel A & B show Similarly, data in Panel, F&G show alpha and beta-diversity results on five human stool samples. Data in Panel H-I represent, Simpson's index, Shannon's entropy and phylogenetic diversity in human stool samples, respectively. Statistical comparisons are presented as Kruskal-Wallis p-values. Beta-diversity was calculated based on Bray-Curtis metric.

Supplementary Figure 4: Abundance of ZymoBIOMICS standard bacteria in different beating times

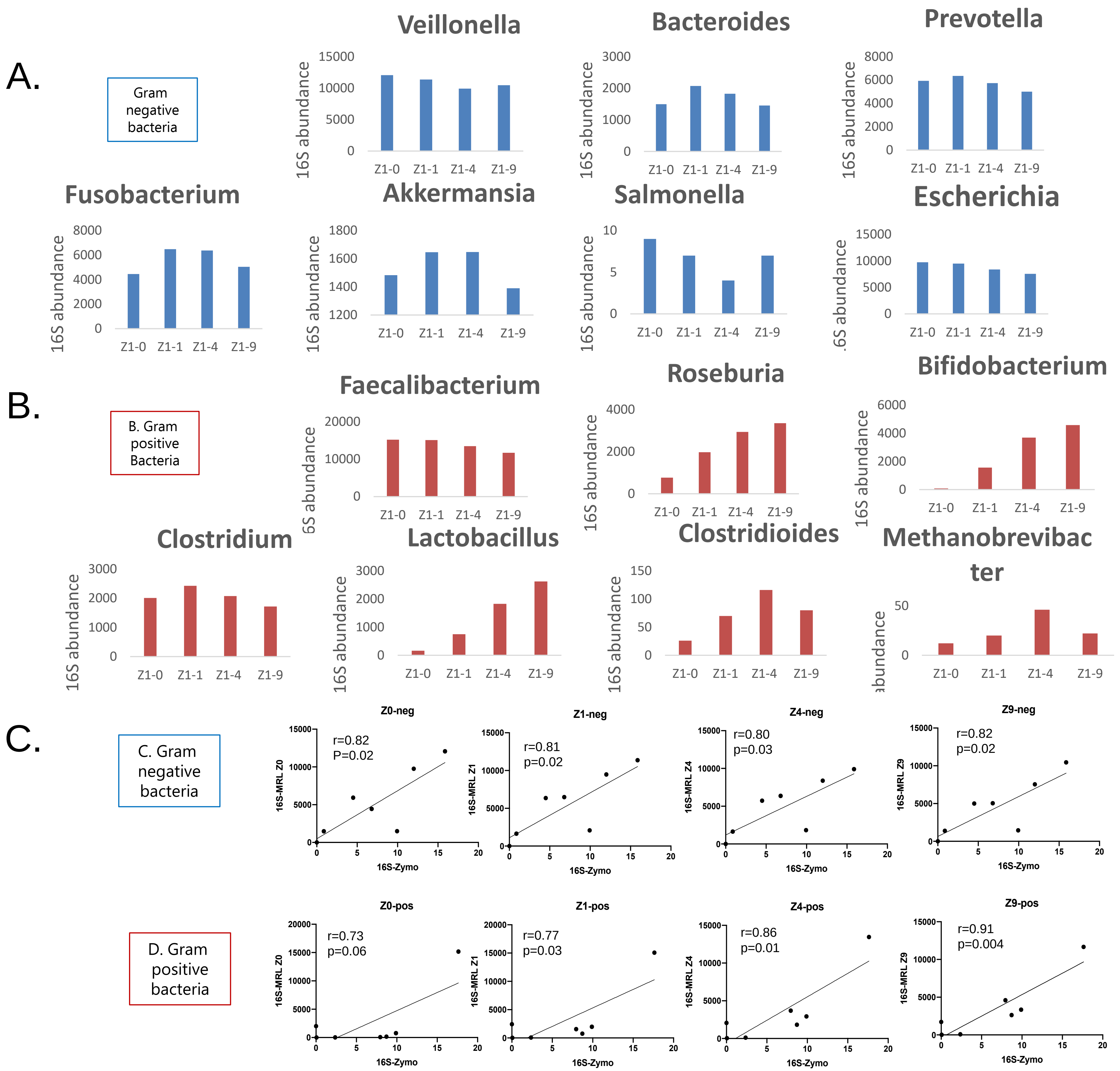

Supplementary Figure 4: Data on ZymoBIOMICS standard bacteria in different beating times based on v3-v4 rRNA amplicon sequencing data. Panel A show abundance of various Gram-negative bacteria in Zymo control at four different bead-beating time treatments. Panel B show abundance of various Gram-positive bacteria in Zymo control at four different bead-beating time treatments. Panel C show correlation between 16 abundances in Zymo Research company's 16S data and present study data. As shown, 9 minutes bead-beating in case of Gram-positive bacteria show maximum correlation. Pearson's correlation analysis was done using GraphPad Prism 8 software.

Supplementary Figure 5: Full length 16S rRNA gene sequencing based high resolution taxonomic classification unravel species diversity in mouse stool

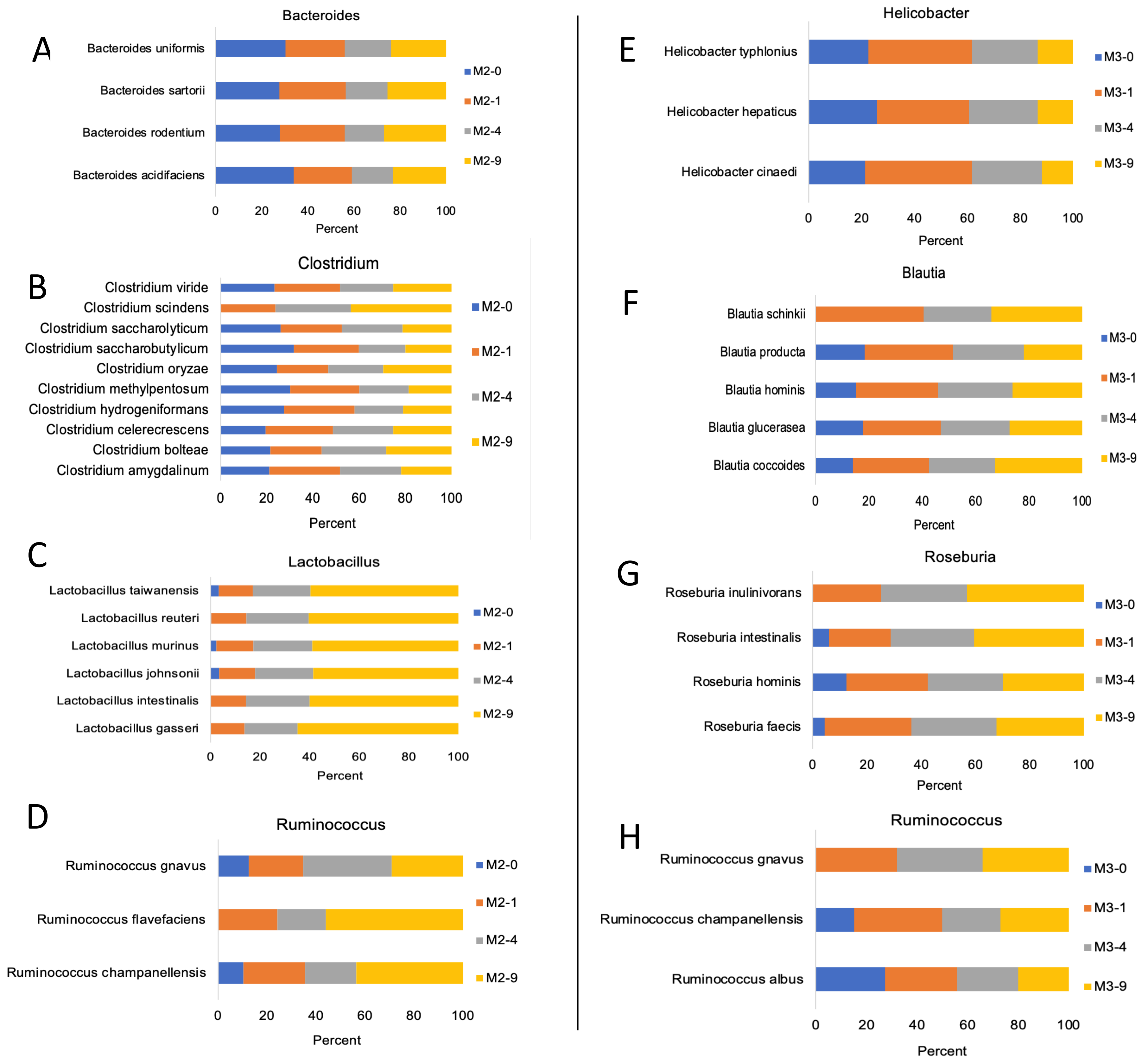

Supplementary Figure 5: Full length 16S rRNA sequencing based taxonomic classification analysis in M2 and M3 moue stool. Panel A-D show abundances of various species of Bacteroides (A), Clostridium (B), Lactobacillus (C) and Ruminococcus (D) in M2 stool at four bead-beating treatment. Panel E-H show abundances of various species in Helicobacter (E), Blautia (F), Roseburia (G) and Ruminococcus (H) in M3 stool at four bead-beating treatment. Percent of reads supporting a given taxon at a given condition were calculated out of total reads observed for that bacteria in total. Those relative percentages are plotted in shown bar graphs.

Supplementary Figure 6: Full length 16S rRNA gene sequencing based high resolution taxonomic classification unravel species diversity in human stool

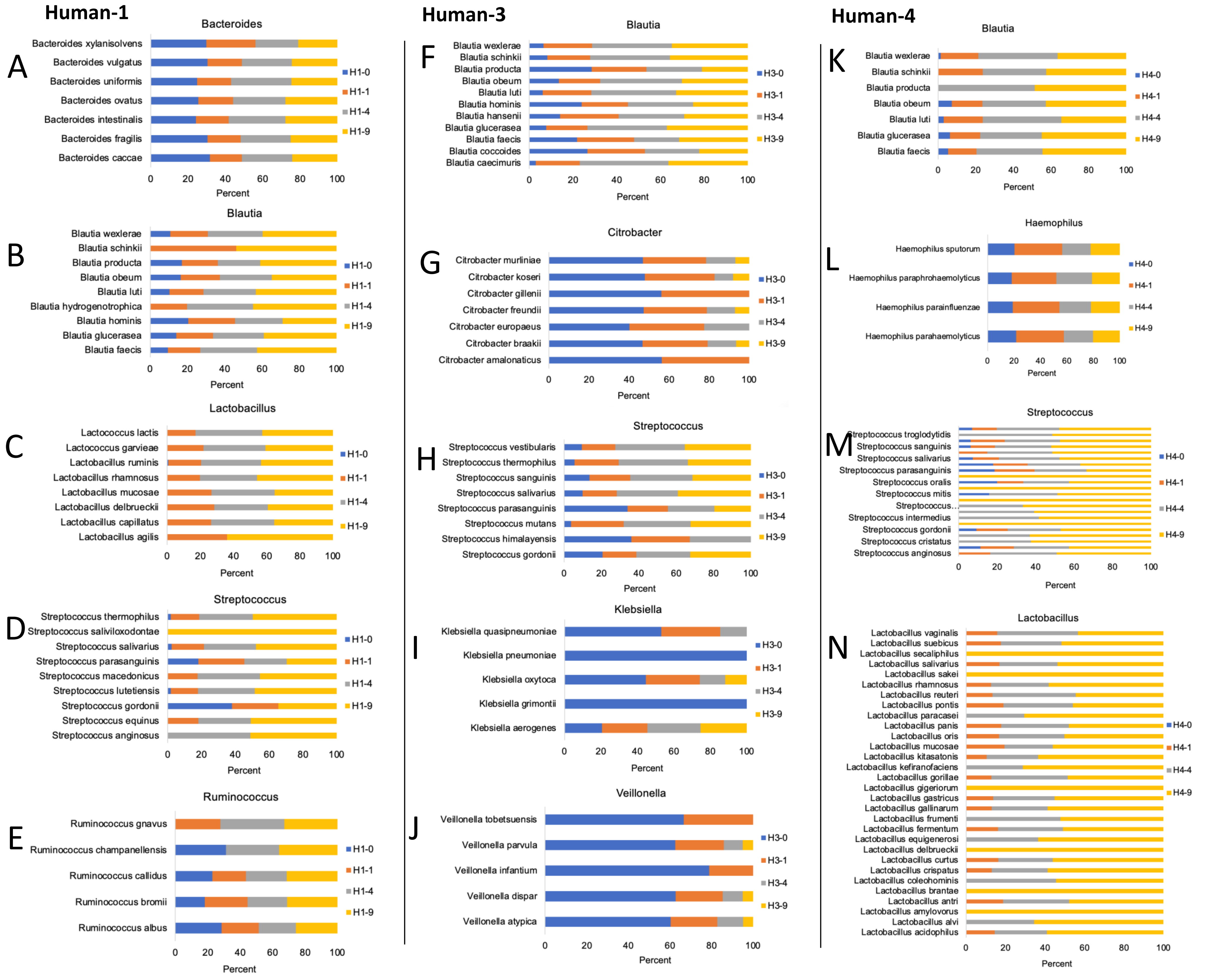

Supplementary Figure 6: Full length 16S rRNA sequencing based taxonomic classification analysis in H1, H3 and H4 human stool. Panel A-E show abundances of various species of Bacteroides (A), Blautia (B), Lactobacillus (C) Streptococcus (D) and Ruminococcus (E) in H1 stool at four bead-beating treatment. Panel F-J show abundances of various species of Blautia (F), Citrobacter (G), Streptococcus (H) Klebsiella (I) and Veillonella in H3 stool at four bead-beating treatment. Panel I-N show abundances of various species of Blautia (K), Haemophilus (L), Streptococcus (M) and Lactobacillus (N) in H4 stool at four bead-beating treatment. Percent of reads supporting a given taxon at a given condition were calculated out of total reads observed for that bacteria in total. Those relative percentages are plotted in shown bar graphs.

Supplementary Figure 7: Stratification of gram stain positive and negative species in H3 human stool based on full length 16S rRNA sequencing data.

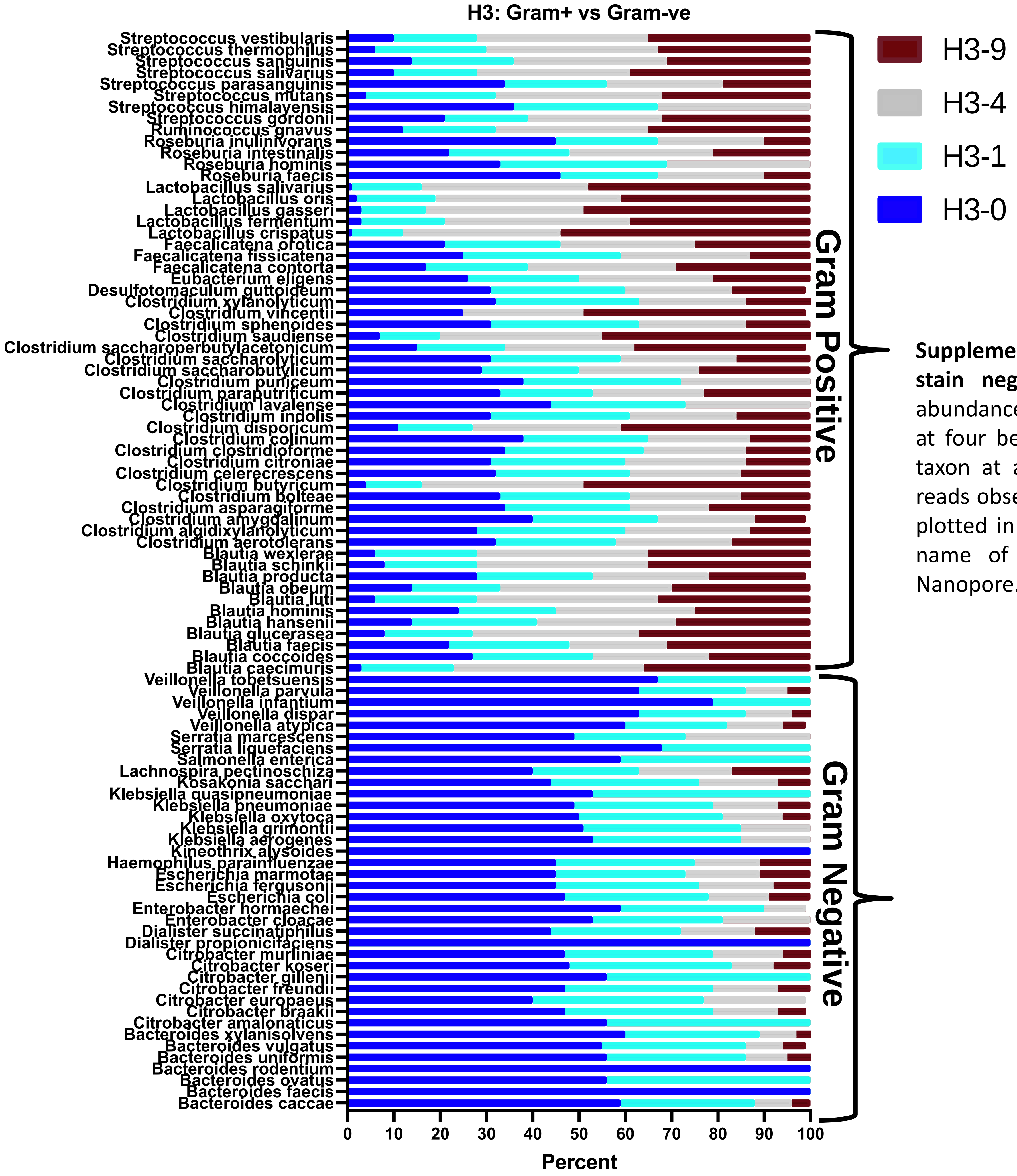

Supplementary Figure 7: Stratification of Gram stain positive and Gram stain negative species in H3 stool sample. Figure show relative abundances of Gram positive (top) and Gram negative (bottom) species at four bead –beating conditions. Percent of reads supporting a given taxon at a given bead-beating condition were calculated out of total reads observed for that bacteria in total. Those relative percentages are plotted in shown bar graphs. X-axis show percentages and Y-axis show name of taxon annotated by EPI2ME WIMP workflow of Oxford Nanopore.

Supplementary Figure 8: Impact of bead beating intensity on the abundance of clinically relevant microbes in zymo mock control and human stool samples

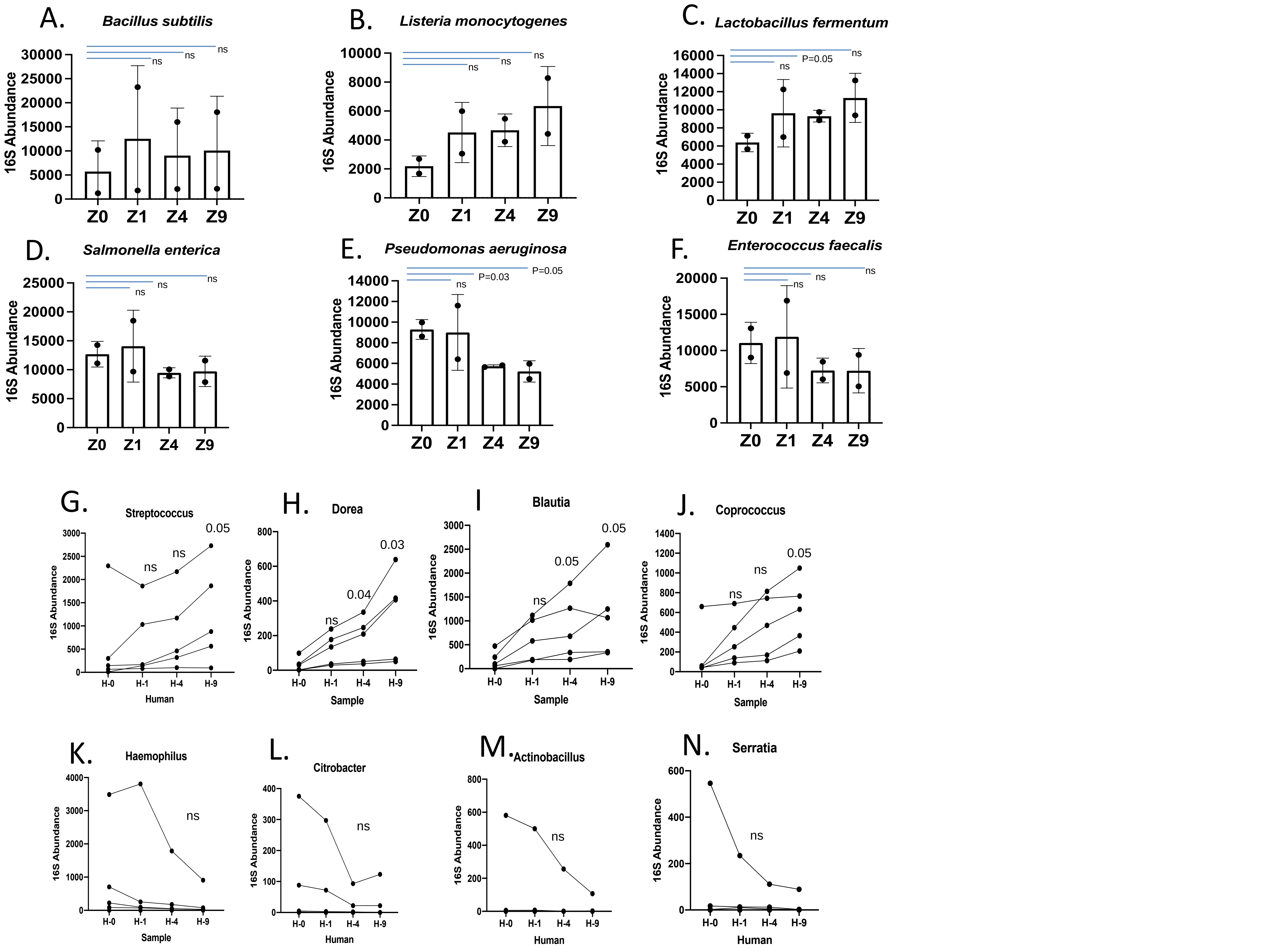

Supplementary Figure 8: Impact of bead-beating on abundances of human disease associated pathogens was assessed in v3-v4 amplicon-based analysis. Panel A-F show the abundance 6 common bacterial species in zymo mock control sample. Labels on X-axis Z0, Z1, Z4 and Z9 represent Zymo control samples treated for 0, 1, 4 & 9 minute of bead-beating , respectively. Y-axis show 16s v3-v4 amplicon data based relative abundances of OTUs. Panel G-J show 16s v3-v4 amplicon sequences derived OTUs in human stool. Data in G-J human disease implicated genera that show higher abundance upon 4 or 9 minutes of bead-beating. Panel K-N show genera that show an opposite trend of higher abundance in un-beaten samples. Each time point was compared using t-test statistics and “ns” indicates “non-significant “ p value.
